# Differential relationships between language skills and working memory in Turkish–Dutch and native-Dutch first-graders from low-income families

**DOI:** 10.1007/s11145-017-9760-2

**Published:** 2017-06-23

**Authors:** Anna M. T. Bosman, Marije Janssen

**Affiliations:** 1 Department of Special Education, Behavioural Science Institute, Radboud University, Montessorilaan 3, 6525 HR Nijmegen, The Netherlands; 2 Flex College, Nijmegen, The Netherlands

**Keywords:** Language skills, Working memory, 1st and 2nd language learners, Bilingualism

## Abstract

In the Netherlands, Turkish–Dutch children constitute a substantial group of children who learn to speak Dutch at the age of four after they learned to speak Turkish. These children are generally academically less successful. Academic success appears to be affected by both language proficiency and working memory skill. The goal of this study was to investigate the relationship between language skills and working memory in Turkish–Dutch and native-Dutch children from low-income families. The findings revealed reduced Dutch language and Dutch working-memory skills for Turkish–Dutch children compared to native-Dutch children. Working memory in native-Dutch children was unrelated to their language skills, whereas in Turkish–Dutch children strong correlations were found both between Turkish language skills and Turkish working-memory performance and between Dutch language skills and Dutch working-memory performance. Reduced language proficiencies and reduced working-memory skills appear to manifest itself in strong relationships between working memory and language skills in Turkish–Dutch children. The findings seem to indicate that limited verbal working-memory and language deficiencies in bilingual children may have reciprocal effects that strongly warrants adequate language education.

## Introduction

The majority of first- and second-generation immigrants in the Netherlands is bilingual. The largest group is from Turkish origin (384,000; the entire Dutch population constitutes 16.5 million people; Centraal Bureau voor de Statistiek, 2008). The first language children in this community learn is the language of their parents, which is predominantly Turkish. Substantial and prolonged exposure to Dutch as a second language usually begins when the Turkish–Dutch children enter kindergarten. This often results in a large group of children from minority backgrounds entering preschool with insufficient knowledge of the Dutch language. A large national-cohort study revealed that children from low-income and minority families start primary school with a delay in their cognitive and Dutch language development of about one standard deviation relative to the average of middle to high-income native-Dutch children (Tesser & Iedema, 2001). The effect is that they cannot benefit optimally from formal education in reading, spelling, and mathematics (Elzer, 2005; van Elten, 2003), shown by the fact that Turkish–Dutch children repeat grades twice as often as native-Dutch children from a similar socio-economic background (Aarts, de Ruiter, & Verhoeven, 1996).

Being a non-native speaker may thus be a disadvantage as shown by the on average, smaller vocabularies of bilinguals in each language compared to monolinguals (Bialystok & Luk, 2012; Pearson, 2007; Scheele, Leeseman, & Mayo, 2010). Differences in vocabulary may appear early and may increase over time. Moreover, differences in vocabulary are found to affect educational achievement in the long run (Baker, Simons, & Kameenui, 1995; Oller & Eilers, 2002; Pearson, Fernandez, Lewedeg, & Oller, 1997).

Although large-scale studies in the Netherlands mostly reveal disadvantages for bilingually raised children, Bialystok (2009) has shown that there may also be advantages of being bilingual, particularly with respect to metalinguistic, cognitive, and conceptual processing, as well as with respect to executive attention and control skills (see also, Yang, Yang, & Lust, 2011). After all, bilingual children need to learn two grammatical systems and must be able to keep the two systems apart. They have to decide which language to use, which words, and which syntactic structure. These skills require highly developed executive functions such as attention shifting and inhibition, functions that are responsible for the control of cognitive processes in working memory

Substantial evidence for this hypothesis comes from Bialystok and her colleagues (e.g., Bialystok, 2002; Morales, Calvo, & Bialystok, 2013; Poulin-Dubois, Blaye, Coutya, & Bialystok, 2011) as well as from other studies. For example, the polyglots of Papagno and Vallar (1995) had superior Italian short-term memory skills than the bilingual control group. Kormi-Nouri et al. (2008) studying Persian monolingual, Turkish–Persian bilingual, and Kurdish-Persian bilingual children (aged 9–10, 13–14, and 16–17 years) showed that the two groups of bilingual children performed better on various types of Persian, episodic- and semantic-memory tasks than monolingual children. This effect was stronger for older bilingual children than for younger ones.

Kormi-Nouri, Moniri, and Nilsson (2003) studied Swedish–Persian bilingual children from a middle-class socioeconomic background and found a positive relationship between bilingualism and episodic and semantic memory. The bilingual children outperformed the monolingual children. This effect was stronger for older bilingual children than for younger ones. The children from this study used their first and second language on a daily basis. The authors concluded that cognitive advantages of being bilingual develop when both languages are mastered to a high proficiency. Da Fontoura and Siegel (1995) showed that Portuguese-English Canadian bilingual children did not differ from monolingual children in reading, syntactic skills, and working memory when language proficiency was equal for both languages. Thus, a high proficiency in both languages may lead to advantages or at least equal performance compared to monolinguals in cognitive and academic skills (Bialystok, 2001; Cummins, 2000).

Other studies, however, have not been able to demonstrate a bilingual memory advantage. Engel de Abreu (2011) tested working-memory skills of a group of 6–8 years old, middle to upper class bilingual children, living in Luxembourg, with Luxembourgish as their second language and those of monolingual Luxembourgish-speaking children. The two groups had similar performance when tested in Luxembourgish. Similarly, for the comparison between 8-year-old bilingual children, from low-income backgrounds, living in Luxembourg whose first language was Portuguese with Luxembourgish as their second language and monolingual Portuguese-speaking children living in Portugal. When tested in their first language no performance differences emerged between the language groups on working memory (Engel de Abreu, Cruz-Santos, Tourinho, Martin, & Bialystok, 2012). Finally, English memory skills of lower-class Portuguese English-speaking bilingual children in Canada did not differ from those of monolingual English-speaking children (Da Fontoura & Siegel, 1995).

Why is it that some studies report a clear advantage of being bilingual, whereas others do not. One possible explanation has been provided by Bialystok (2001). She argued that a bilingual advantage with respect to working memory is more likely to occur when there is a high proficiency in the two languages (see also Cummins, 2000). Other factors that may explain bilingual advantage are social class and exposure to both languages. Almost all positive findings with respect to a bilingual advantage pertain to middle- or upper-class children who had been exposed to both languages since their birth and who also used both languages daily. The only exception is the study conducted by Engel de Abreu (2011). Her sample of bilingual children, exposed to both languages from birth and who came from middle to upper class background, did not outperform a control group of monolingual children, albeit the monolingual children scored better than the bilingual children on language measures pertaining to vocabulary and syntax.

All three studies (Da Fontoura & Siegel, 1995; Engel de Abreu, 2011; Engel de Abreu et al., 2012) in which no bilingual advantage was found concerned Portuguese as first or native language and in two of them the participating children came from low-income backgrounds. To our knowledge no other study has been conducted in which working memory was studied in disadvantaged children and related to language skills. The present study attempts to contribute to the knowledge concerning the role of working memory in the development of children who are exposed to their second language long after they started learning their native language; a common situation in (Dutch) immigrant children from poor families. The most important task for the Turkish–Dutch children in the Netherlands is to learn an additional (i.e., second) language with vocabulary acquisition as its major goal.

The reason to emphasize memory is that young children need to remember an ever-increasing number of new words and Sternberg (1987), along with many others (e.g., de Jong, 1998; Siegel & Ryan, 1989; St Clair Thompson & Gathercole, 2006), have claimed that the single most important factor for successful intellectual and educational development is vocabulary acquisition. In their seminal paper, Baddeley, Gathercole, and Papagno (1998) proposed that the primary function of the phonological loop, an essential component of Baddeley and Hitch’s working-memory model (Baddeley and Hitch, 1994, see Baddeley, 2000 for an adaptation), is “…to provide a mechanism for the temporary storage of new words while more stable long-term phonological representations are being constructed.” (p. 166, Baddeley et al., 1998). Baddeley et al. assume a causal connection between the phonological loop and vocabulary learning. In an earlier paper, Gathercole, Willis, Emslie, and Baddeley (1992) found evidence for this assumption using a cross-lagged correlation design.

Baddeley and Hitch’s working-memory model is a three-component system with a so-called attention-control system known as the ‘central executive’ with two subsidiary systems, namely, the ‘phonological loop’ and the ‘visuospatial sketchpad’, holding verbal and acoustic information, and visuospatial information, respectively, in a temporary store. In line with earlier research, we focused on the phonological loop, because of its role in keeping information in store by rehearsing it and on the central executive, because of its role in maintaining as well as manipulating information (this system is responsible for the control of cognitive processes). Measures that are assumed to assess the phonological loop and the central executive, rather than the visuospatial sketchpad have been shown to be good predictors of language skills (e.g., Gathercole & Baddeley, 1993; Messer, Leseman, Boom, & Mayo, 2010; Papagno & Vallar, 1995).

An important and mostly unstated assumption in the literature on second language acquisition is that working memory is an innate, largely unchangeable, individual cognitive characteristic affecting native and foreign language acquisition and performance (for recent overviews, see Linck, Osthus, Koet, & Bunting, 2014; Wen, Borges Mota & McNeill, 2015). Robust and positive relationships between working memory and second/foreign language proficiency have been reported extensively, emphasizing the importance of working memory in learning a new language.

In this study, three types of tasks were used to assess verbal-working memory: Digit recall, backward-digit recall, and listening recall. Digit recall only takes into account the storage function, whereas the complex tasks (listening recall and backward-digit recall) also account for the processing functions. In accordance with the model of Baddeley and Hitch (1994, 2000; see also Diamond, 2013 for a more recent discussion), digit recall measures the short-term storage capacity of the phonological loop, backward-digit recall and listening recall include also the central executive.

A final issue that will be addressed in this paper is the language in which working-memory will be tested. In most studies, working memory is either tested in the native language or in the second language. Because of a unique situation in the Netherlands with respect to the Turkish language, it was possible to test language skills as well as assess working memory in both Dutch and Turkish.

The aim of the present study is twofold. The first goal is to investigate the relationship between language skills and verbal-working memory within the two language groups. As said, the relationship between language development and working memory in bilingual children from low-income background has not been widely studied and they may well differ from those of middle- or high-income children. A second goal is to compare working-memory skills of bilingual Turkish–Dutch children with those of monolingual native-Dutch children, from low-income families in the Netherlands to investigate the effect of learning a second language only upon entering school. Although Baddeley et al. (1998) assumed a causal connection between the phonological-loop component of working memory and vocabulary development, the design we used was not suitable to test this supposal. The following questions guided our investigation and reveal correlations rather than causations:

1. Are Dutch language skills of bilingual Turkish–Dutch children from low-income backgrounds indeed lower than that of native-Dutch children?
2. Is Turkish still better developed than Dutch in first-grade Turkish–Dutch children, and what is the relationship between Dutch and Turkish language skills in Turkish–Dutch children?
3. Is there a difference between Dutch working memory of Turkish–Dutch children and native-Dutch children?
4. Do Turkish–Dutch children perform better on Turkish working-memory tasks than on Dutch working-memory tasks?
5. To what extent are Dutch language skills related to Dutch working-memory skills, and is this different for Turkish–Dutch children and native-Dutch children?
6. To what extent are Turkish language skills related to Turkish working-memory skills in Turkish–Dutch children?

## Method

### Participants

In this study participated 38 Turkish–Dutch (24 boys, 14 girls, *M* age = 7;4, *SD* = 0.6) and 48 (25 boys, 23 girls; *M* age = 7;2, *SD* = 0.6) native-Dutch children who all attended first grade during testing. All children were recruited from the same inner-city neighborhoods populated by low-income and immigrant families (i.e., all families had a low socio-economic status). All Turkish–Dutch children were born in the Netherlands; the majority of their parents (95% of fathers and mothers) were born in Turkey. All Turkish–Dutch children learned Turkish as their first language and for the majority of them (65%) this language was still the best developed by the age of three. Almost all children (80%) were to some extent exposed to Dutch as a second language before the age of three by watching Dutch television or playing with Dutch-speaking children, including older siblings who already attended Dutch-primary schools. Nonetheless, starting preschool meant for most of them a strong increase in Dutch-language input. The native-Dutch children were born in the Netherlands and grew up in low-income families and only spoke Dutch, and thus are considered monolingual. When they were 3 years old, all Turkish–Dutch as well as the native-Dutch children attended a preschool program for disadvantaged children, because of their limited Dutch language proficiency (a center-based program to enhance language skills and socio-emotional development).

To assess general language and cognitive differences between the two groups, a language-comprehension test (i.e., Reynell test for language comprehension by van Eldik, lutje Spelberg, Schlichting, van der Meulen, & van der Meulen, 1997) and a nonverbal-intelligence test (i.e., the Standard Progressive Matrices or SPM; Raven, 1958) were administered. There were no intellectual differences between the two groups (*F* < 1; Turkish–Dutch children: *M* = 25.0, *SD* = 7.5; native-Dutch children: *M* = 27.7, *SD* = 6.2) as assessed by the SPM. With respect to language comprehension, however, the native-Dutch children (*M* = 71.0, *SD* = 8.9) outperformed the Turkish–Dutch children (*M* = 78.9, *SD* = 4.1), *F*(1, 51) = 10.89, *p* = .002.

### Materials

To measure the language skills of the two language groups two subtests of the diagnostic test of Bilingualism (i.e., vocabulary and sentence imitation) were used, developed by the National Institute of Educational Testing (Cito; Verhoeven, Narain, Extra, Konak, & Zerrouk, 1995). All children were tested in Dutch, and the Turkish-speaking children were also tested in Turkish.

#### Vocabulary

The productive-vocabulary test consists of 40 pictures. Children were presented with a picture book displaying one picture per page. The children had to answer the question “What is that?” or “What happens here?” A correct answer was rewarded with one point. When a child failed five consecutive items the test was ended. The minimum score was 0 and the maximum score was 40. The Dutch and Turkish versions of the active-vocabulary test are highly reliable with Cronbach’s alpha being .90 and 85, respectively.

#### Sentence-imitation task

This test measures syntactic knowledge. Children were orally presented with 20 sentences, one by one, and asked to repeat each sentence as accurately as possible. For each sentence, the correct reproduction of two distinct grammatical structures was scored: function words and word order. The mean sentence length and mean number of nominal and verbal phrases were the same in the two languages. The minimum score was 0 and the maximum sore was 40; scores were converted into percentages. The Dutch and Turkish sentence-imitation tests are highly reliable with Cronbach’s alpha being .95 for both tests.

To measure verbal-working memory, three subtests of the Automated Working Memory Assessment Battery (AWMA; Alloway, 2007) were adapted for Dutch and Turkish by Messer et al. (2010). The AWMA can be used to test children between the ages of 4.5 and 11.5 years. Each test begins with a series of practice trials immediately followed by the test trials. The test ends when three or more errors within a block of trials were made. The score for that test reflects the number of correct responses up to the point at which the test was ended; scores were converted into percentages.

#### Digit recall

The child had to repeat a sequence of voiced digits (1–9) in the same order as presented. The test started with a block of one digit and ended with a block of nine digits. The test consisted of 54 items divided in nine blocks of six trials each that increased in difficulty. Each correct trial was rewarded with one point. The minimum score was 0 and the maximum score was 54; scores were converted into percentages. For the Turkish–Dutch children, parallel digit-span tests in both Dutch and Turkish, using the count words from 1 to 9 in Turkish, were administered on two different occasions within a 2-months’ period.

#### Listening recall

The child listened to a series of sentences and had to judge whether a sentence was true or false, for instance “lions have legs and tomatoes play football.” At the same time the child had to memorize the first word of each sentence. After all sentences were presented and evaluated, the child had to recall each first word of each sentence, in the same order as presented. The sentences were presented in growing set sizes starting with a one-sentence trial and ending with a six-sentence trial. The entire test consisted of 36 items divided in six blocks of six trials that increased in difficulty. Each correct trial was rewarded with one point. The minimum score was 0 and the maximum score was 36; scores were converted into percentages. In the original test of Alloway, Gathercole, and Pickering (2006), children had to remember the last word of each sentence. We changed this to the first word to accommodate for the fact that the Turkish language has a verb-last structure (OSV, SOV). Turkish sentences usually start with a subject or object, a content word with a clear lexical meaning. Parallel versions were developed for the Turkish and Dutch tests, such that the first word of each sentence to be remembered was always a content word. There is a caveat to that. In cross-linguistic research, equating the difficulty of materials is always a challenge, hence direct comparison of parallel tasks in Turkish and Dutch has to be interpreted cautiously.

#### Backward-digit recall

The child had to repeat a sequence of spoken digits (1–9) in reverse order. The test started with a block of two digits and ended with a block of seven digits. The test consisted of 36 items divided in six blocks of six trials that increased in difficulty. Each correct trial was rewarded with one point. The minimum score was 0 and the maximum score was 36; scores were converted into percentages. The Turkish–Dutch children were given Turkish and Dutch parallel tests.

Psychometric characteristics of the Dutch version of the working-memory tests could not be computed, because only total scores were collected. Fortunately, Alloway, Gathercole, and Pickering (2006) assessed the psychometric characteristics of the English tests and they proved satisfactory. Of course, we realize that results from studies in the English language do not prove unequivocally that a similar set-up in Dutch is therefore satisfactory.

Another indication of the reliability measures of the Dutch and Turkish versions of the AWMA listening-recall test were assessed in the doctoral research project of Messer (2010). Measures taken at ages 5 and 6 in a sample of Dutch and Turkish–Dutch children showed moderate stability over a 1-year interval (*r* = .45, *p* < .001) for the Dutch version (*n* = 136) and similarly for the Turkish version (*r* = .54, *p* < .001, *n* = 65), indicating sufficient test–retest reliability. Concurrent correlations between listening recall and backward digit recall for Dutch (*r* = .49, *p* < .001) and for Turkish (*r* = .31, *p* < .01) and between listening recall and a visual-spatial task for Dutch (*r* = .36, *p* < .001) and for Turkish (*r* = .14, not significant) appeared to be unsatisfactory.

### Procedure

All children were tested individually in a quiet room in their school between February and May. Tasks were presented in a fixed order. First the children were tested in Dutch by a non-Turkish speaking Dutch person, 2 months later a native-speaking Turkish researcher tested the Turkish–Dutch children in their mother tongue on all three working-memory tests as well as on vocabulary and sentence imitation.

## Results

The results’ section is divided in three parts. The first and second section present the results of the language tests and working-memory tests, respectively, examining the differences between Turkish–Dutch and native-Dutch children on these skills. In the third section the relationships between the working-memory tests and the language tests are presented.

### Language skills

Prior to the analyses, all dependent variables were tested on normalcy by means of the Shapiro–Wilk test. The Dutch tests revealed significant deviations from normalcy for vocabulary (*W* = .95, 68, *p* < .001) and sentence imitation (*W* = .91, *p* < .005). We therefore decided to use Mann–Whitney to test non-parametrically for the differences between groups. The first columns of Table 1 present the descriptive statistics of each group on both tests in Dutch. Native-Dutch children outperformed the Turkish–Dutch children on Dutch-productive vocabulary (*U* = 116.0, *n1* = 47, *n2* = 21, *p* < .001) and Dutch-sentence imitation (*U* = 69.0, *n1* = 16, *n2* = 21, *p* < .002).

**Table 1:** Means and standard deviations in percentages correct on vocabulary and sentence imitation in Dutch and in Turkish (for Turkish–Dutch children only)

	Dutch language		Turkish language	
	Vocabulary	Sentence imitation	Vocabulary	Sentence imitation
Turkish–Dutch children				
Mean	48.6	71.3	38.3	44.7
*SD*	12.2	15.7	17.0	19.6
*n*	21	21	24	22
Native-Dutch children				
Mean	68.8	84.8	–	–
*SD*	11.6	14.9	–	–
*n*	47	16		

Next, performance of the Turkish–Dutch children on the Dutch-language tests was compared with their performance on the Turkish-language tests using Wilcoxon Signed Rank tests for paired samples. The last columns of Table 1 present the descriptive statistics of Turkish–Dutch children on the Turkish and Dutch versions of productive vocabulary and sentence imitation. Turkish–Dutch children were better on Dutch-productive vocabulary than on Turkish-productive vocabulary (*Z* = 2.22, *p* < .03) and better on Dutch-sentence imitation than on Turkish-sentence imitation (*Z* = 3.10, *p* < .002).

Spearman non-parametric correlations were computed to assess relationships among and between language skills. Table 2 presents the correlations. The figures show that the language skills of the native-Dutch children did not correlate significantly, whereas those of the Turkish–Dutch children revealed significant and high correlations between Dutch-productive vocabulary and Dutch-sentence imitation and between Turkish-productive vocabulary and Turkish-sentence imitation. There were no significant relationships between Dutch and Turkish vocabulary or between Dutch-sentence and Turkish-sentence imitation.

**Table 2:** Spearman correlations between and among Dutch and Turkish language tests

	Language group	
	Turkish–Dutch	Native-Dutch
Dutch vocabulary × Dutch sentence imitation		
*rho*	.72***	−.10
*n*	21	16
Turkish vocabulary × Turkish sentence imitation		
*rho*	.57**	
*n*	22	
Dutch vocabulary × Turkish vocabulary		
*rho*	.23	
*n*	18	
Dutch sentence imitation × Turkish sentence imitation		
*rho*	.009	
*n*	16	

** *p* < .01, *** *p* < .0001

To summarize, these findings reveal that Dutch-language skills of native-Dutch children were better than those of Turkish–Dutch children. Turkish–Dutch children had better Dutch-language skills than Turkish-language skills. Dutch-productive vocabulary and Dutch-sentence imitation were unrelated skills in native-Dutch children, but highly related in Turkish–Dutch children (Fischer *Z* = 2.11, *p* = .01). Turkish-productive vocabulary and Turkish-sentence imitation were also highly related, but Turkish vocabulary and Dutch vocabulary or Turkish-sentence imitation and Dutch-sentence imitation were not.

### Working memory

First the results of the working-memory tests of the Turkish–Dutch and the native-Dutch children on the Dutch version of the working-memory tests will be presented. Second, a comparison will be made for the Turkish–Dutch children only between achievements in their first language, Turkish, and their second language, Dutch.

#### Monolingual versus bilingual children on Dutch working-memory tests

Shapiro–Wilk tests revealed significant deviations from normalcy for Dutch-listening recall (*W* = .97, *p* < 0.02) and Dutch backward-digit recall (*W* = .95, *p* < 0.002); the distribution of Dutch digit-recall was normally distributed (*p* = 0.27). We therefore decided to use Mann–Whitney to test non-parametrically for the differences between groups. Table 3 presents the descriptive statistics for each group regarding the mean percentage of correct items on all three Dutch working-memory tests. Native-Dutch children outperformed the Turkish–Dutch children on all three tests: digit recall (*U* = 522.5, *n1* = 48, *n2* = 38, *p* = .001); listening recall (*U* = 536.5, *n1* = 48, *n2* = 38, *p* = .001); backward-digit recall (*U* = 678, *n1* = 48, *n2* = 38, *p* = 0.04).

**Table 3:** Means and standard deviations of percentage correct of the Dutch working memory tests of the two participant samples

	Working-memory tests		
	Digit recall	Listening recall	Backward digit recall
Turkish–Dutch children			
Mean	43.2	18.9	20.8
*SD*	5.9	11.4	13.2
*n*	38	38	38
Native-Dutch children			
Mean	48.1	26.5	25.8
*SD*	6.3	9.6	6.8
*n*	48	48	48

Analyses pertaining to performance differences on the three Dutch working-memory tests were conducted for the two groups separately, using Friedman. There was a significant effect of type of working-memory test in the native-Dutch group, *X* ^2^(2) = 69.7, *p* < .001. Post-hoc Wilcoxon Signed Rank tests for paired samples revealed that performance on digit recall was significantly better than listening recall (*Z* = 5.95, *p* < .001) and backward-digit recall (*Z* = 6.03, *p* < .001). No significant difference emerged between listening recall and backward-digit recall (*p* = .79).

The same analyses for the Turkish–Dutch group revealed a similar pattern, *X* ^2^(2) = 57.7, *p* < .001. Post-hoc Wilcoxon Signed Rank tests for paired samples revealed that performance on digit recall was significantly better than listening recall (*Z* = 5.38, *p* < .001) and backward-digit recall (*Z* = 5.36, *p* < .001). No significant difference emerged between listening recall and backward-digit recall (*p* = .50).

#### Bilingual children on Dutch and Turkish working-memory tests

Shapiro–Wilk tests revealed no significant deviations from normalcy for Turkish digit recall (*p* = 0.17), listening recall (*p* = 0.36) or Turkish backward-digit recall (*p* = 0.08). Because the Dutch working-memory tests were not normally distributed, we decided to run non-parametric Wilcoxon Signed Rank tests for paired samples. Table 4 presents the descriptive statistics of each working-memory test in both languages. The difference between the Dutch and Turkish scores on the digit recall was significant (*Z* = 3.51, *p* < .001), whereas those on listening recall (*p* = .58) and backward-digit recall (*p* = .46) were not.

**Table 4:** Means and standard deviations of percentages correct on working memory of Turkish–Dutch children tested in both Dutch and Turkish

	Digit recall	Listening recall	Backward digit recall
Dutch language			
Mean	44.7	20.4	23.5
*SD*	5.1	9.4	12.0
*n*	38	38	38
Turkish language			
Mean	50.3	18.2	25.1
*SD*	7.6	11.6	12.2
*n*	27	26	25

The means of the Dutch working-memory tests presented in this table deviate slightly from those in Table 3, because not all Turkish–Dutch children who were administered the Dutch tests received the Turkish version

* *p* < .01

A comparison of the Turkish working-memory tests yielded a significant difference, *X* ^2^(2) = 35.5, *p* < .001. Post-hoc Wilcoxon Signed Rank tests for paired samples revealed that performance on digit recall was significantly better than listening recall (*Z* = 4.46, *p* < .001) and backward-digit recall (*Z* = 4.29, *p* < .001), and backward-digit recall was significantly better than listening recall (*Z* = 2.01, *p* < .001).

Spearman non-parametric correlations between the Turkish and Dutch versions of each of the three working-memory tests were also conducted and revealed that Turkish digit recall and Dutch digit recall did not correlate significantly (*rho* = .28), similarly for listening recall (*rho* = .33), whereas Turkish and Dutch backward-digit recall did (*rho* = .53, *p* < .01).

To summarize, the native-Dutch children outperformed the Turkish–Dutch children on all three Dutch working-memory tests. Turkish–Dutch children performed better on digit recall when tested in Turkish than in Dutch, but they obtained similar scores for listening recall and backward-digit recall in Turkish and Dutch. Dutch Digit recall was easier than listening recall and backward-digit recall for both groups, listening recall and backward-digit recall was equally difficult. With respect to the Turkish working-memory tests, digit recall was easier than backward-digit recall, which in turn was easier than listening recall. Interestingly, only a strong performance association between the Turkish and Dutch versions of backward-digit recall was apparent.

### The relationships between working memory and language skills

Spearman non-parametric correlations were computed between the three Dutch tests for working memory (i.e., digit recall, listening recall, and backward-digit recall) and the two Dutch language tests (i.e., productive vocabulary and sentence imitation) for the native-Dutch children and for the Turkish–Dutch children. The findings are presented in Table 5.

**Table 5:** Spearman correlations between all three tests of Dutch working memory and the two Dutch language tests for both language groups

Working-memory test	Vocabulary		Sentence imitation	
	Turkish–Dutch children (*n* = 21)	Native-Dutch children (*n* = 47)	Turkish–Dutch children (*n* = 21)	Native-Dutch children (*n* = 16)
Digit recall				
*rho*	.69***	.09	.55**	.30
Fischer *Z*	2.71, *p* = .01		.99, *p* = .32	
Listening recall				
*rho*	.42*	.08	.57**	.51*
Fischer *Z*	1.31, *p* = .19		0.23, *p* = .82	
Backward digit recall				
*rho*	.42	−.01	.23	.45
Fischer *Z*	1.64, *p* = .10		−0.69, *p* = .49	

* *p* < .05, ** *p* < .01, *** *p* < .0001

One of the correlations of the native-Dutch sample reached significance, namely the one between sentence imitation and listening recall, whereas four out of six correlations reached substantial and significant levels in the Turkish–Dutch sample. To substantiate the differences between the two language groups, Fischer-*Z* tests were conducted on the six correlational comparisons. One comparison reached significance, namely, the correlation between vocabulary and digit recall was larger in the Turkish–Dutch group than in the native-Dutch group.

Finally, Spearman correlations were computed between the three Turkish tests for working memory and the two Turkish language tests for the Turkish–Dutch children. Turkish-productive vocabulary as well as Turkish-sentence imitation is significantly related with Turkish digit recall and Turkish listening recall, but not with Turkish backward-digit recall (see Table 6).

**Table 6:** Spearman correlations between all three tests of Turkish working memory and the two Turkish language tests for the Turkish–Dutch children only

Working memory	Vocabulary	Sentence imitation
Digit recall		
*rho*	.41*	.53**
* n*	23	21
Listening recall		
* rho*	.65**	.55**
* n*	22	20
Backward digit recall		
* rho*	.35	.42
*n*	23	21

* *p* < .05, ** *p* < .01, *** *p* < .0001

To summarize, these findings revealed that Dutch working-memory skills are largely unrelated to Dutch language skills in the native-Dutch children, but in four out of six cases they were related in Turkish–Dutch children. Difference in correlational strength between the language groups was only apparent with respect to the association between vocabulary and digit recall. Also, the Turkish working-memory skills of digit recall and listening recall were strongly related to Turkish language skills in the Turkish–Dutch children.

## Discussion

The goal of this study was twofold. One, compare working memory skills between bilingual Turkish–Dutch children and monolingual native-Dutch children from low-income families in the Netherlands. Two, establish the relationships between language performance and working memory within each language group. The answers to the six questions stated in the introduction will provide the background for addressing the main goals of the present study.

### Language skills

Native-Dutch children had, as expected, better Dutch language skills than Turkish–Dutch children, both with respect to vocabulary and sentence imitation. An interesting result was that the Turkish–Dutch children performed better on the Dutch language tests than on the Turkish ones. At the age of six or seven, Turkish–Dutch children have certainly acquired a great deal of knowledge regarding the Dutch language, but their skills are not yet at the level of those of native-Dutch children from similar backgrounds. The fact that performance on Turkish tests was below that of Dutch tests suggests that the development of Turkish in this group is slowing down. The children from this Turkish background only hear Turkish at home or in the family. Unlike in the past, these children are unable to attend Turkish lessons, because financing of extracurricular language education for children who are non-native speakers of Dutch was stopped some years ago.

Another important finding was the differential relationship between performance on Dutch vocabulary and Dutch-sentence imitation of the two language groups. Performance on these tasks was unrelated in native-Dutch children, but highly related in Turkish–Dutch children as was Turkish vocabulary and Turkish-sentence imitation. Also, in the group with more limited language skills relationships between different language tasks was strong; a finding we will return to below.

### Working memory

Native-Dutch children outperformed the Turkish–Dutch children on all three Dutch memory tasks. Thus, children with the better language skills also had a better memory performance. Both language groups scored better on digit recall, a task tapping in the storage function of memory than on listening recall and backward-digit recall, tasks that refers storage as well as processing or manipulating information in memory.

Turkish-memory performance was only superior on digit recall; the Turkish–Dutch children performed equally well on Dutch-listening recall and Turkish-listening recall and on Dutch backward-digit recall and Turkish backward-digit recall. Storage of Turkish numbers appears to be the only aspect of memory that it is still better developed than Dutch numbers in Turkish–Dutch children, which converges with the earlier finding that Turkish–Dutch children were performing worse on the Dutch digit-recall task. The manipulation of information in memory is equally well developed in Dutch and Turkish. In an earlier study, Janssen, Bosman, and Leseman (2013) showed that Dutch phoneme awareness of Turkish–Dutch children in a comparable sample was better than in Turkish. The work of Da Fontoura and Siegel (1995) presented an opportunity to assess performance difference on working-memory tasks in first and second language of a group of Portuguese-English bilingual children. Irrespective of their reading level, all children performed better on the English version of the working-memory task than on the Portuguese one (see Note 1, for the statistical analyses). Because it is impossible to compare the level of proficiency of the participants of the present study with that of Da Fontoura and Siegel (1995), it may be worthwhile to conduct a comparative study that will shed some more light on the development of memory and language in bilingual children.

Note also that a strong performance association emerged between the Dutch and Turkish version of the backward-digit recall task, no such correlations existed for digit recall and listening recall. Performance on backward-digit recall was worse than on digit recall but equally good on listening recall.

### Relationship between memory and language skills

With respect to associations between language and memory skills, an interesting pattern emerged: Dutch-working memory and Dutch language are unrelated skills in native-Dutch children, but highly related in Turkish–Dutch children. Turkish-working memory and Turkish-language skills are also highly related skills in Turkish–Dutch children. These findings combined with the inferior language skills of the Turkish–Dutch children suggest that a minimal level of language development is required to strengthen verbal-working memory skills. Stated differently, limited exposure to language input, suggests that experience with a particular language (i.e., Dutch) determines, at least partly, the capacity of verbal-working memory in that language.

Sufficient semantic and syntactic knowledge required to support the capacity of verbal-working memory in listening recall in the Turkish–Dutch group, may not have been sufficiently developed yet, neither in Turkish nor in Dutch. Turkish–Dutch children’s performance on the Turkish sentence-imitation task indicated low-syntactic sensitivity in Turkish, which was even lower than their syntactic sensitivity in Dutch. Being exposed to a second language, that is the dominant language in society after development of the first language has started, offers a possible explanation. Listening recall requires good language proficiency. Languages proficiency provides options for chunking and integrating verbal (semantically and syntactically structured) information, as is especially needed in performing listening-recall tasks (Service, 1992). A language delay may therefore limit verbal-working memory and may slow down language acquisition (Messer et al., 2010; Thorn & Frankish, 2005).

Strong associations between skills seem to indicate that skills have not yet fully developed. An example from the reading literature reveals that phonological skills are strongly related to reading performance, but only at the onset of reading development. After children gain reading experience the correlation between phonological skills and reading drops and usually even disappears (e.g., de Jong & van der Leij, 2002; Furness, & Samuelsson, 2009; Patel, Snowling, & de Jong, 2004). Thus, after children become more proficient in skills, the initial relationship between performances diminishes over time.

Our findings are in accordance with a longitudinal study of Gathercole et al. (1992) with monolingual English-speaking children from predominantly middle-class homes. They found that phonological memory becomes increasingly unimportant with increasing age (between 4 and 8 years). In our native-Dutch sample (mean age of 7 years), the relationship between working-memory and language skills was also non-existent. However, the strong relationship between language and memory skills in the Turkish–Dutch children who were lagging behind in both language and memory development appear to show a pattern that mimics that of native speakers at an earlier age and as such coincides with the strong relationships found in Gathercole et al.’s sample of younger native speakers between the ages of 4 and 6.

Gathercole et al. (1992) provided a couple of reasons for the diminishing effect of phonologic constraints on language development (i.e., vocabulary acquisition). One is that with growing age, children’s vocabulary may profit from the analogies with already acquired words, which may in turn take of the burden of phonological memory. Another reason is that it becomes increasingly important to acquire the meaning of more abstract words, which might reduce the effect of phonological memory. Finally, the effect that reading has on learning new words may well outweigh the contribution of phonological memory.

As a final remark on the relationship between working memory and language, the cross-lagged design used by Gathercole et al. (1992) indeed provides an interesting perspective on the directionality of this relationship, but it does not decisively rule out the possibility of a reciprocal relationship. We would like to entertain this thought when we reflect upon the implications for practice.

### Implications

Suppose, the strong association between language skills and verbal-working memory in children with limited language skills are in fact a sign of reciprocal effects in a developmental process of mutually constituting abilities (see also Jones, Gobet, & Pine, 2008; Messer et al., 2010). This perspective then has a number of implications and points to an important task for school teachers. First of all, children, who as a consequence of being raised bilingually and growing up in a language poor environment are lagging behind in the language used in the school, face a double problem. Not only are they disadvantaged in school language as such, but probably also in the ability to learn school language from the input provided at school. Moreover, this effect may easily spread to several subject areas involving understanding instruction, learning verbally-stated knowledge, and reading comprehension. Several studies, indeed, indicated that poor working memory is a predictor of persistent learning difficulties in several school subjects. Gathercole, Alloway, Willis, and Adams (2006) conclude that “working memory acts as a bottleneck for learning.” (p. 278). Gathercole (2008) observed children with poor working memory to have more difficulties in following instructions, keeping place in a complex task, coping with simultaneous storage and processing demands, and longer-term remembering. Given the tendency in the literature to view working memory as a domain-general ability which is hardly dependent on experience and instruction (Swanson, 2001), the present study adds an important new perspective to these analyses, namely that a limited verbal working-memory, may—at least partly—be caused by language deficiencies that could be remediated by supporting language development at an early age. Working memory may after all be subject to change (i.e., improvement) rather than being viewed upon as an innate cognitive capacity that should be taken as a given.

## Note

(1) Table 2 of Da Fontoura and Siegel (1995) presents the mean scores for a group of normally achieving readers (*n* = 24) on Portuguese working memory (5.1, *SD* = 1.7) and on English working memory (6.0, *SD* = 2.1). The authors did not present the statistics, but a *t* test for dependent samples revealed a significant difference, *t*(23) = −2.1, *p* < 0.05. A similar computation for a group of reading disabled children also proved to be significant, *t*(11) = −2.3, *p* < 0.05; Portuguese working memory (3.7, *SD* = 1.5) and English working memory (4.9, *SD* = 1.8).
